# Functional Intestinal Bile Acid 7α-Dehydroxylation by *Clostridium scindens* Associated with Protection from *Clostridium difficile* Infection in a Gnotobiotic Mouse Model

**DOI:** 10.3389/fcimb.2016.00191

**Published:** 2016-12-20

**Authors:** Nicolas Studer, Lyne Desharnais, Markus Beutler, Sandrine Brugiroux, Miguel A. Terrazos, Laure Menin, Christian M. Schürch, Kathy D. McCoy, Sarah A. Kuehne, Nigel P. Minton, Bärbel Stecher, Rizlan Bernier-Latmani, Siegfried Hapfelmeier

**Affiliations:** ^1^Institute for Infectious Diseases, University of BernBern, Switzerland; ^2^Graduate School for Cellular and Biomedical Sciences, University of BernBern, Switzerland; ^3^Environmental Microbiology Laboratory, École Polytechnique Fédérale de Lausanne (EPFL)Lausanne, Switzerland; ^4^Max von Pettenkofer Institute of Hygiene and Medical Microbiology, Ludwig-Maximilians-University of Munich (LMU)Munich, Germany; ^5^Institute of Chemical Sciences and Engineering, École Polytechnique Fédérale de Lausanne (EPFL)Lausanne, Switzerland; ^6^Institute of PathologyUniversity of Bern, Bern, Switzerland; ^7^Maurice Müller Laboratories (DKF), Universitätsklinik für Viszerale Chirurgie und Medizin Inselspital, University of BernBern, Switzerland; ^8^Clostridia Research Group, Biotechnology and Biological Sciences Research Council (BBSRC) and the Engineering and Physical Sciences Research Council (EPSRC) Synthetic Biology Research Centre, School of Life Sciences, University of NottinghamNottingham, UK; ^9^German Center for Infection Research, Deutsches Zentrum für Infektionsforschung (DZIF), Partner Site Ludwig-Maximilians-University of Munich (LMU)Munich, Germany

**Keywords:** 7α-dehydroxylation, *Clostridium scindens*, *Clostridium difficile*, gnotobiotic mouse model, secondary bile acids, gut microbiota, *Clostridium difficile* infection (CDI), intestinal infection

## Abstract

Bile acids, important mediators of lipid absorption, also act as hormone-like regulators and as antimicrobial molecules. In all these functions their potency is modulated by a variety of chemical modifications catalyzed by bacteria of the healthy gut microbiota, generating a complex variety of secondary bile acids. Intestinal commensal organisms are well-adapted to normal concentrations of bile acids in the gut. In contrast, physiological concentrations of the various intestinal bile acid species play an important role in the resistance to intestinal colonization by pathogens such as *Clostridium difficile*. Antibiotic therapy can perturb the gut microbiota and thereby impair the production of protective secondary bile acids. The most important bile acid transformation is 7α-dehydroxylation, producing deoxycholic acid (DCA) and lithocholic acid (LCA). The enzymatic pathway carrying out 7α-dehydroxylation is restricted to a narrow phylogenetic group of commensal bacteria, the best-characterized of which is *Clostridium scindens*. Like many other intestinal commensal species, 7-dehydroxylating bacteria are understudied *in vivo*. Conventional animals contain variable and uncharacterized indigenous 7α-dehydroxylating organisms that cannot be selectively removed, making controlled colonization with a specific strain in the context of an undisturbed microbiota unfeasible. In the present study, we used a recently established, standardized gnotobiotic mouse model that is stably associated with a simplified murine 12-species “oligo-mouse microbiota” (Oligo-MM^12^). It is representative of the major murine intestinal bacterial phyla, but is deficient for 7α-dehydroxylation. We find that the Oligo-MM^12^ consortium carries out bile acid deconjugation, a prerequisite for 7α-dehydroxylation, and confers no resistance to *C. difficile* infection (CDI). Amendment of Oligo-MM^12^ with *C. scindens* normalized the large intestinal bile acid composition by reconstituting 7α-dehydroxylation. These changes had only minor effects on the composition of the native Oligo-MM^12^, but significantly decreased early large intestinal *C. difficile* colonization and pathogenesis. The delayed pathogenesis of *C. difficile* in *C. scindens*-colonized mice was associated with breakdown of cecal microbial bile acid transformation.

## Introduction

Bile acids are biologically important cholesterol derivatives produced in the liver and secreted into the bile as glycine- or taurine-conjugated primary bile acids (Chiang, 2009). The main primary bile acids produced are cholic acid (CA) and chenodeoxycholic acid (CDCA) in humans, and CA and muricholic acid (MCA) in mice (Hofmann, 1999; Russell, 2003). The conjugated forms of these primary bile acids are released through the bile duct into the duodenum, where they act as detergents for the emulsification and absorption of dietary lipids (Hofmann, 2009). Beyond their digestive function, bile acids also act as hormone-like regulators of host metabolism (Kuipers et al., 2014) as well as antimicrobials (Begley et al., 2005). Their potency is influenced by several microbial transformations during intestinal transit.

In the small intestine, primary bile acids are deconjugated by bile acid hydrolases which are expressed by a wide variety of intestinal bacterial taxa including species belonging to the *Bacteroidetes* (Narushima et al., 2006), a dominant group within the gut microbiota. About 95% of the primary bile acids are reabsorbed in the small intestine and recycled via the enterohepatic circulation (Russell, 2003; Ridlon et al., 2005; Hofmann, 2009). The remaining 5% that escape the enterohepatic circulation undergo further microbial transformations in the large intestine (Ridlon et al., 2005, 2016). One of the most important bile acid transformations is 7α-dehydroxylation, generating secondary bile acids, including deoxycholic acid (DCA) and lithocholic acid (LCA). This multistep biochemical pathway, encoded in the *bai* (“bile acid inducible”) gene operon, is restricted to a narrow phylogenetic group of bacterial species belonging to the *Clostridium* cluster XIV and has been most extensively studied in the species *Clostridium scindens* and *Clostridium hylemonae* (Ridlon et al., 2005, 2016).

Transformation of host-produced conjugated primary bile acids to DCA and LCA is an example of microbial inter-species co-metabolism. 7α-dehydroxylation bacteria depend on other microbial species for deconjugation of the glycine- or taurine-conjugated bile acids, as they can only metabolize unconjugated bile acids. For this reason, bile acid 7α-dehydroxylation by human 7α-dehydroxylating species *C. scindens* and *C. hylemonae* cannot occur in mono-colonized animals, in absence of bile acid deconjugating organisms (Narushima et al., 1999).

Secondary bile acids play a key role in resistance to intestinal infection (Begley et al., 2005). The best-studied example is *Clostridium difficile* infection (CDI), a nosocomial, antibiotic therapy-associated diarrheal infection (Rupnik et al., 2009). Resistance against CDI conferred by a healthy gut microbiota is abolished when perturbed (dysbiosis) by broad-spectrum antibiotic treatment (Britton and Young, 2012). The antibiotic-induced dysbiosis is associated with the abolition of secondary bile acid biosynthesis in the colon (Antunes et al., 2011; Theriot et al., 2016, 2014).

It is well-established that bile acids regulate *C. difficile* endospore germination and outgrowth as well as vegetative growth (Wilson et al., 1982; Wilson, 1983). Whilst the germination receptors of *C. difficile* are only partially identified (Francis et al., 2013a; Fimlaid et al., 2015), the primary bile acids taurocholic acid (TCA) and cholic acid as well as the secondary bile acid DCA are proven germinants of *C. difficile* spores (Sorg and Sonenshein, 2008). Moreover, chenodeoxycholic acid (CDCA) as well as alpha and beta stereoisomers of muricholic acid (MCA) are known to inhibit *C. difficile* germination (Sorg and Sonenshein, 2010; Francis et al., 2013b). Further, DCA as well as LCA, ursodeoxycholic acid (UDCA) and ω-muricholic acid (ω-MCA) are strong inhibitors of spore outgrowth and vegetative cell growth of *C. difficile* (Francis et al., 2013b; Weingarden et al., 2015). Thus, it has been hypothesized that bile acid deconjugating and 7α-dehydroxylating microbial species play a critical role in conferring colonization resistance to *C. difficile*. Initial evidence supporting this hypothesis was mainly derived from *in vitro* experiments; direct evidence for the importance of microbial bile acid transformation in CDI *in vivo* is still scarce. Only recently, Buffie and colleagues, by using metagenomic analyses and computational modeling of antibiotic-treated animal and patient samples, were able to infer a strong positive correlation between the presence of *C. scindens*-related taxa and CDI resistance, and successfully tested causality *in vivo* by inoculation of antibiotic-treated, CDI-susceptible mice with the *C. scindens* type strain ATCC35704 (Buffie et al., 2014).

The intestinal microbiology of *C. scindens* and related species is still understudied. A limitation of previous work has been that *C. scindens* colonization experiments *in vivo* required antibiotic-treated animals containing a dysbiotic microbiota with unclear background levels of endogenous bile acid 7-dehydroxylating bacteria. Although this situation mimics the conditions in the CDI patient, it can be an experimental limitation for the study of *C. scindens* physiology in the healthy gut.

Whilst it is standard to use genetically defined inbred mouse strains for most experimental animal studies, conventional animal models contain still rudimentarily defined, and unstandardized microbial consortia. These consist of hundreds to thousands of different operational taxonomic units and vary considerably between individual animals as well as among research facilities (Rogers et al., 2014; Ericsson et al., 2015; Hoy et al., 2015). Although modern analytical and computational tools facilitate the analysis of complex host–microbiota interactions, it is a challenge to establish the underlying signaling and metabolic networks without the ability to standardize also the microbiota composition. Experimental animal models with a fully standardized microbiota, so-called gnotobiotic animal models, provide a defined and reduced complexity *in vivo* system for the in-depth elucidation of host–microbiota interactions.

In the present study, we used a recently established defined mouse intestinal microbial consortium, referred to as “oligo-mouse microbiota” (Oligo-MM^12^), rationally assembled of 12 mouse-intestinal bacterial isolates representative of the major mammalian intestinal bacterial phyla (Brugiroux et al., 2016) as a platform for the *in vivo* study of *C. scindens*. The bacterial strains combined in the Oligo-MM^12^ consortium were recently described in detail and are openly available from the DSMZ repository (Lagkouvardos et al., 2016). Gnotobiotic Oligo-MM^12^-associated mice (henceforth referred to as “stable defined moderately diverse microbiota, murine 2,” short sDMDMm2 mice) were originally generated by colonization of germ-free mice with a cocktail of cultured bacteria. They have been stably maintained by breeding under gnotobiotic conditions for over 25 generations in three independent facilities without significant compositional deviations, providing mice harboring a physiologically acquired, and developed stable gut microbiota.

We amended the Oligo-MM^12^ microbiota by colonization of gnotobiotic sDMDMm2 mice with *C. scindens* ATCC35704 and assayed the resulting bile acid 7α-dehydroxylation activity and protection against CDI *in vivo*. For this purpose we first compared the CDI-susceptibility of sDMDMm2 mice with germ-free and antibiotic treatment mouse models of CDI and carried out a comprehensive quantitative analysis of the associated bile acid metabolomes. We then analyzed the effects of the introduction of *C. scindens* into the preexisting stable sDMDMm2 consortium. We show that *C. scindens* strain ATCC35704 can be functionally amended to sDMDMm2 to carry out CA and CDCA 7α-dehydroxylation. In correlation with this finding, *C. scindens* colonization specifically delayed intestinal overgrowth of *C. difficile* and concomitant intestinal pathology. Only minor direct effects of *C. scindens* on the composition of the 12 species of the Oligo-MM^12^ model consortium were detectable.

## Materials and methods

### Animals and Oligo-MM^12^ microbiota

Groups of age matched C57BL/6 mice (6–12 weeks old) were used. Group sizes used in each experiments are indicated in the figure legends. Germ-free and gnotobiotic sDMDMm2 mice (Brugiroux et al., 2016) were established and maintained at the Clean Mouse Facility (CMF) of the Department of Clinical Research of the University of Bern. sDMDMm2 mice were colonized with a mouse-intestine derived 12-species mouse microbiota (Oligo-MM^12^) consisting of the following bacterial species: *Acutalibacter muris* sp. nov. KB18 (DSM 26090), *Flavonifractor plautii* YL31 (DSM26117), *Clostridium clostridioforme* YL32 (DSM 26114), *Blautia coccoides* YL58 (DSM 26115), *Clostridium innocuum* I46 (DSM26113), *Lactobacillus reuteri* I49 (DSM 32035), *Enterococcus faecalis* KB1 (DSM 32036), *Bacteroides caecimuris* sp. nov. I48 (DSM 26085), *Muribaculum intestinale* sp. nov. YL27 (DSM 28989), *Bifidobacterium longum* subsp. *animalis* YL2 (DSM 26074), *Turicimonas muris* sp. nov. YL45 (DSM 26109) and *Akkermansia muciniphila* YL44 (DSM 26127). All Oligo-MM^12^ strains are available at http://www.dsmz.de/miBC. In-depth description of the Oligo-MM^12^ consortium species and description of novel taxa are provided elsewhere (Brugiroux et al., 2016; Lagkouvardos et al., 2016). SPF mice were purchased from Envigo (formerly Harlan, The Netherlands). Germ-free and sDMDMm2 mice were exported from flexible film breeding isolators into sterile individually ventilated cages (IVC; model Sealsafe Plus, Tecniplast, Italy) for *C. difficile* infection experiments. All animal experiments were performed in accordance with the Swiss Federal and the Bernese Cantonal regulations and were approved by the Bernese Cantonal ethical committee for animal experiments under the license number BE 82/13.

### Bacterial strains and culture conditions

*C. scindens* ATCC35704 (Morris et al., 1985) was grown in BHIS+ medium, which contains 37 g BHI, 5 g yeast extract, 40 ml salts solution (0.2 g CaCl_2_, 0.2 g MgSO_4_, 1 g K_2_HPO_4_, 1 g KH_2_PO_4_, 10 g NaHCO_3,_ and 2 g NaCl in 1 L _dd_H_2_O), 1 g L-cysteine and 2 g fructose per L _dd_H_2_O or on BHIS+ agar. Erythromycin (10 μg/mL) was added to BHIS+ medium to select for *C. scindens* ATCC35704 from fecal/cecal samples. *C. difficile* DH1916 (Burns et al., 2011) was grown in BHIS medium, BHIS agar or *Clostridium difficile* fructose agar (CDFA). Erythromycin (10 μg/mL), cycloserine (0.5 mg/mL), cefoxitin (16 μg/mL), and taurocholate (0.1%) were added to plates where necessary. BHIS+ plates for the enumeration of *C. scindens* were incubated in an anaerobic workstation (Don Whitley A45 HEPA, 10% CO_2_, 10% H_2_, 80% N_2_) for 72 h at 37°C. CDFA or BHIS plates for the enumeration of *C. difficile* were incubated in anaerobic jars using an atmospheric generator (Genbox anaer, bioMérieux, France) for 24–48 h at 37°C or in the anaerobic workstation for 24–48 h at 37°C.

### Preparation of *C. difficile* spores

*C. difficile* DH1916 was grown overnight in BHIS medium. A 100 μL aliquot of the overnight culture was plated onto BHIS agar plates and incubated for 5 days in an anaerobic workstation at 37°C. Plates were removed from the workstation and stored at 4°C overnight. Using a loop, all cell material was scraped into 1 mL _dd_H_2_O (three plates per mL). The spore suspensions were re-incubated at 4°C overnight to free spores from mother cells and subsequently washed for at least 10 times in ice-cold _dd_H_2_O. Spore preps were controlled for their purity using phase contrast microscopy and spores were enumerated using a hemocytometer as well as by plating on BHIS plates containing 0.1% taurocholic acid. All spore preparations were heat-treated (65°C, 30 min) to ensure complete inactivation of any remaining vegetative cells. All Spore preps were stored at 4°C until use.

### Pretreatments and infection with *C. difficile*

SPF mice were antibiotic-pretreated with Clindamycin (100 μL by intraperitoneal injection, 2 mg/mL in PBS) or Streptomycin (100 μL by gavage, 200 mg/mL in PBS) 24 h before infection. sDMDMm2 mice were inoculated with *C. scindens* ATCC35704 by gavage of 10^9^–10^10^ CFU *C. scindens* and colonized for 4 or 7 days before *C. difficile* infection or sacrifice, as specified. *C. scindens* pre-colonization was performed in flexible film isolators, and the mice were subsequently exported into sterile IVCs for *C. difficile* infection. *C. difficile* DH1916 was administered by gavage of 10^3^ CFU of spores suspended in 100 μL sterile PBS.

### Lipocalin-2 quantification

Lipocalin-2 was measured from terminally collected cecal content using a commercial ELISA kit (R&D Systems, USA). The wells of a 96-well plate were coated with lipocalin-2 capture antibody (1:200 in PBS, 50 μL, incubated at 4°C overnight), washed with wash buffer (0.05% Tween-20 in PBS) and blocked with 150 μL blocking buffer (2% BSA in PBS, 3 h at RT). The diluted samples (10-fold dilutions over four wells) and the standards (duplicate, 3-fold dilutions over eight wells) were applied and the plates were incubated at 4°C overnight. After washing with wash solution, 100 μL of biotinylated anti-lipocalin-2 antibody was applied (1:200 in blocking buffer, 50 μL) and incubated for 1 h at room temperature. The wells were washed with wash buffer and treated with horseradish-peroxidase-streptavidin (Biolegend, USA, 1:1000 in PBS, 100 μL) for 1 h at room temperature. Development was performed using 100 μL of substrate buffer (0.1 M NaH_2_PO_4_, pH 4.0) with 0.01% ABTS and 0.1% H_2_O_2_. The absorbance at 415 nm was measured with a plate reader. Standards and samples were fitted using a non-linear regression and lipocalin-2 values were calculated back to ng of lipocalin-2 per g of sample.

### Histopathology

Cecal tissue was fixed in 4% PFA/PBS immediately after the sacrifice of the mice and kept at 4°C for 24 h. The cecum tissue was rehydrated using a 20% Sucrose/PBS solution at 4°C for at least 24 h. The ceca were embedded in Optimal Cutting Temperature (OCT) medium (Tissue-Tek, Sakura, Japan) and stored at −80°C. Sections of 7 μm thickness were cut, air dried and stained with hematoxylin, and eosin. Cecal histopathology was evaluated in a blinded manner by a pathologist using an established scoring system for acute intestinal inflammation as described (Barthel et al., 2003). Scoring criteria were: (1) Submucosal edema; 0 = no pathological changes, 1 = mild edema; the submucosa is <0.20 mm wide, 2 = moderate edema; the submucosa is 0.21 to 0.45 mm wide, 3 = profound edema; the submucosa is >0.46 mm wide. (2) Polymorphonuclear cell (PMN) infiltration into the lamina propria; PMN were enumerated in 10 high-power fields (400x magnification in a field diameter of 420 μm) and the average number of PMN/high-power field was calculated, 0 ≤ 5 PMN/high-power field, 1 = 5–20 PMN/high-power field, 2 = 21–60 PMN/high-power field, 4 ≥ 100 PMN/high-power field. (3) Loss of goblet cells; Goblet cells were enumerated in 10 high-power fields (400x magnification in a field diameter of 420 μm) of cecum epithelium and the average of Goblet cells per high-power field was calculated, 1 = 11–28 goblet cells/high-power field, 2 = 1–10 goblet cells/ high-power field, 3 ≤ 1 goblet cell/ high-power field. (4) Epithelial integrity; 0 = no pathological changes, 1 = epithelial desquamation, 2 = erosion of the epithelial surface (gaps of 1–10 epithelial cells per lesion), 3 = epithelial ulceration (gaps of >10 epithelial cells per lesion). The combined histopathological score was determined as the sum of these individual scores.

### Calprotectin RT-qPCR

To measure the expression of calprotectin, cecal tissue was taken at the time of necropsy. The cecal tissue was emptied from all fecal matter, washed in sterile PBS, and immediately stored in RNAlater (Qiagen, Germany). RNA was extracted from the tissue using the RNEasy kit (Qiagen, Germany) according to the manufacturer's instructions. RNA quality was analyzed with the Agilent Bioanalyzer system using RNA microchips (Agilent Technologies, USA). Amplification of calprotectin and the housekeeping gene β-actin was performed using the following primers and probes: calprotectin probe (CTC TGC TAC TCC TTG TGG CTG TCT), calprotectin fw (AGT GTC CTC AGT TTG TGC AGA), calprotectin rev (CAG GGA CCC AGC CCT AG), β-actin probe (CCA GTT GGT AAC AAT GCC ATG TTC), β-actin fw (TAG GCA CCA GGG TGT GAT G), β-actin rev (TGC CAG ATC TTC TCC ATG TCG). Both probes were labeled with 6-carboxyfluorescein (FAM) on the 5′ and the Black hole quencher (BHQ) on the 3′ end. All probes were synthesized and tested by Microsynth (Switzerland) according to MIQE guidelines (Bustin et al., 2009). RT-PCR was performed in duplicates in a volume of 20 μL using the TaqMan RNA to C_t_ 1-Step kit (Applied Biosystems, USA) on a QuantStudio 7 Flex Real-Time PCR System (Thermo Fisher, USA) with the following program: 15 min 48°C, 10 min 95°C followed by 40 cycles of 15 s 95°C, and 1 min 60°C. Fluorescence was measured for each cycle after then step of 60°C. C_t_-values for both genes were averaged over both measurements and the mean C_t_ of calprotectin was subtracted from the mean C_t_-value of β-actin to normalize.

### Bile acid extraction

Deuterated CDCA and deoxycholic acid were used as recovery standards. Approximately 10 milligrams of freeze-dried cecal content was homogenized with 150 μL of H_2_O and spiked with 20 μL of recovery standards (100 μM), using an automated Precellys 24 bead-based homogenizer (5000 rpm; 1 × 20 s). Mixed samples were equilibrated on ice for 1 h. An amount of 500 μL of ice-cold alkaline (5% ammonia in ACN) was added to the homogenate, which was then vigorously vortexed, and continuously shaken for 1 h at room temperature. The mixture was centrifuged at 16,000 g for 10 min and the supernatant collected. The pellet was extracted with another 500 μL of ice-cold alkaline ACN. Supernatants from the two extractions steps were pooled and dried in a rotational vacuum concentrator (Christ, Germany) before reconstitution in 100 μL of 50:50 ammonium acetate (5 mM): methanol (MeOH) pH 6. The supernatant was kept at −20°C until LC-MS injection.

### UHPLC-HRMS analyses

Both qualitative and quantitative analyses were conducted on a Agilent 6530 Accurate-Mass Q-TOF LC/MS mass spectrometer coupled to an Agilent 1290 series UHPLC system (Agilent Technologies, USA). The separation was achieved using a Zorbax Eclipse-Plus C18 column (2.1 × 100 mm, 1.8 μm; Agilent Technologies, USA) heated at 50°C. A binary gradient system consisted of 5 mM ammonium acetate pH 6 in water as eluent A and acetonitrile as eluent B. The sample separation was carried out at 0.4 mL/min over a 19 min total run time using the following program: 0–7 min, isocratic 21% B; 7–7.5 min, 21–25% B; 7.5–11.5 min, 24–30% B; 11.5–14.5 min, 30–32% B; 14.5–17.5 min, 32–63% B; 17.5–19 min, 63–100% B. The system was reequilibrated in initial conditions for 2 min. The sample manager system temperature was maintained at 4°C and the injection volume was 0.5 μL. Mass spectrometer detection was operated in negative ionization mode using the Dual AJS Jet stream ESI Assembly. The QTOF instrument was operated in the 4 GHz high-resolution mode (typical resolution 17,000 (FWHM) at *m/z* 1000) in profile mode and calibrated in negative full scan mode using ESI-L solution (Agilent Technologies, USA). Internal calibration was performed during acquisition via continuous infusion of a reference mass solution [5 mM purine, 1 mM HP-921 (Agilent reference mass kit, Agilent Technologies USA) in 95% MeOH acidified with 0.1% formic acid] and allowed to permanently achieve a mass accuracy better than 5 ppm. HR mass spectra were acquired over the range of *m/z* 300–700 at an acquisition rate of three spectra/s. AJS settings were as follows: drying gas flow, 8 L/min; drying gas temperature, 300°C; nebulizer pressure, 35 psi; capillary voltage, 3500 V; nozzle voltage, 1000 V; fragmentor voltage, 175 V; skimmer voltage, 65 V; octopole 1 RF voltage, 750 V. Data was processed using the MassHunter Workstation (Agilent Technologies, USA). According to this method, 36 bile acids (Table S1) were quantified by external calibration curves. Standard stock solutions were prepared at a concentration of 10 mM in methanol. Bile acid solutions were mixed together and diluted with 50:50 ammonium acetate (5 mM):MeOH to construct standard curves between 5 and 5000 nM. Extracted ions chromatograms (XIC) were based on a retention time (RT) window of ±0.5 min with a mass-extraction-windows (MEW) of ±30 ppm centered on *m/z*_*theor*_ of each bile acid.

### Quantitative PCR of bacterial 16S rRNA genes

16S rRNA specific primers and hydrolysis probes were designed using Primer Express 3 (Applied Biosystems, Life Technologies, USA). For duplex qPCR assays, hydrolysis probes were 5′-labeled with either 6-carboxyfluorescein (FAM) or 6-carboxyhexafluorescein (HEX). Each probe was conjugated with the black hole quencher 1 (BHQ1) at the 3′ end. Primers and probes were synthesized by Metabion (Planegg, Germany). qPCR conditions were established according to the MIQE guidelines (Minimum Information for Publication of Quantitative Real-Time PCR Experiments) (Bustin et al., 2009). All primers were designed for an optimal annealing temperature of 60°C. qPCR standard curves were determined using linearized plasmid as DNA template. Plasmid DNA was diluted in H_2_O containing 100 ng/μl yeast tRNA (Roche, Switzerland). Standard curves were run on a Roche Lightcycler96 instrument in triplicates. Efficiency of each qPCR reaction was calculated based on the slope of standard curves (qPCR efficiency: (10^(−1/slope of standard curve)^−1) × 100) using 10-fold dilutions of template. Efficiencies for all qPCR reactions were within the range of 90–110%. For all experiments the software LightCylcer96 version 1.1 reproduced standard curves based on single DNA template with known DNA quantity as well as the efficiency derived from the standard curve of each qPCR assay of the initial run of the standard curves. Specificity was confirmed by performing an assay for each primer/probe pair using an equimolar mixture of all linearized plasmids except for the one to be tested as template. One PCR reaction (total volume: 20 μl) contained 300 nM of each primer, 250 nM of the corresponding hydrolysis probe (Table S2), FastStart Essential DNA Probes Master (Roche, Switzerland), and 5 ng template gDNA. PCR reactions with DNA templates extracted from feces or cecal content were run in duplicates. PCR conditions were: 95°C for 10 min, followed by 45 cycles of 95°C for 15 s, and 60°C for 1 min. Fluorescence for each cycle was recorded after the step at 60°C. Quantification cycle (Cq) as well as the baseline were automatically determined by the software LightCycler96 version 1.1 (Roche, Switzerland). The detection limit ranged between 1 and 248 16S rRNA gene copies/5ng template gDNA (Table S2).

### Statistical analysis

Statistical analyses were performed as described in Figure Legends using GraphPad Prism 6 (Version 6.07) or R (Version 3.1.2). Hierarchical clustering in **Figures 2**, **4** used the dist function (Canberra distance) as well as the hclust function with the “complete” method. The NMDS analysis in **Figure 5** used the function metaMDS of the R package vegan as well as the adonis function for statistical analysis of the differences between groups.

## Results

### CDI susceptibility in microbially undefined and defined mouse models

**Figure 1:**
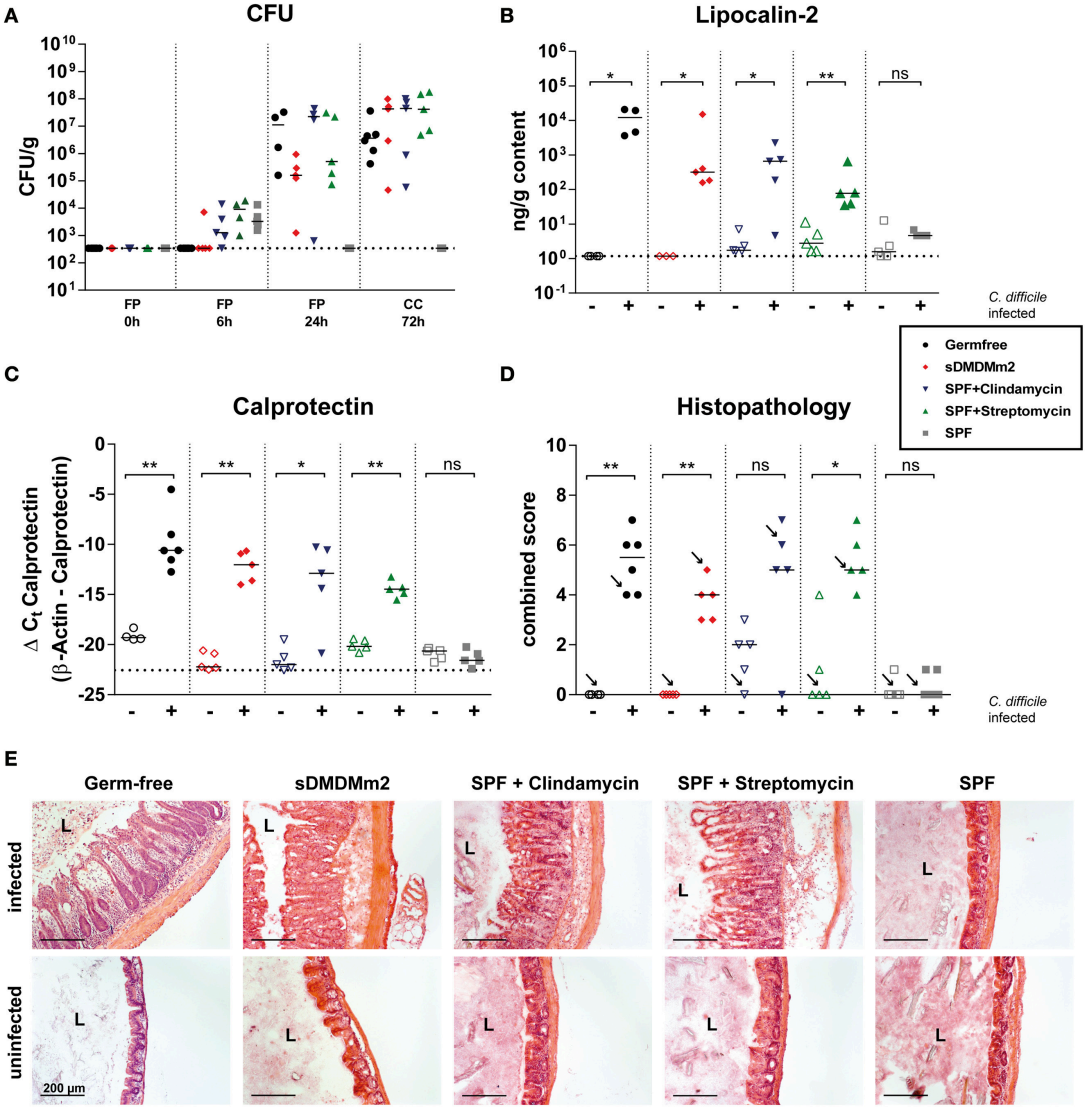
**Infections with ***C. difficile*** in sDMDMm2 mice resemble infections in antibiotic-treated mice**. Cohorts of germ-free (black circles, *n* = 6), sDMDMm2 (red diamonds, *n* = 5), clindamycin-treated SPF (purple inverted triangles, *n* = 5), streptomycin-treated SPF (green triangles, *n* = 5), and SPF control (gray squares, *n* = 5) mice were gavaged with 10^3^ CFU *C. difficile* DH1916 (filled symbols) or PBS vehicle as control (open symbols). **(A)** Comparison of CFU of *C. difficile* in fecal pellets (FP) or cecal contents (CC) at different time points over course of infection. **(B)** Comparison of lipocalin-2 measured by ELISA in cecal content after 72 h of infection. **(C)** Comparison of calprotectin from cecal tissue after 72 h of infection measured by qPCR. Values show the ΔC_t_ derived from measured values of calprotectin with β-actin as control. **(D)** Histopathological evaluation of H&E stained sections of cecum tissue at time of necropsy (72 h). Arrows indicate the representative mice depicted in **(E)**. **(E)** Representative images of H&E stained sections of cecum tissue at time of necropsy (72 h). L, Lumen; Scale bars: 200 μm. Statistical analyses in **(B–D)** used Mann-Whitney-U tests to compare uninfected with infected animals at individual timepoints. Each symbol represents one individual. Bars indicate medians. Dotted lines indicate lower limit of detection. ns, not statistically significant (*p* ≥ 0.05); ^*^p < 0.05; ^**^p < 0.01.

We first addressed the question of whether the sDMDMm2 mice colonized with the 12-species Oligo-MM^12^ consortium (Brugiroux et al., 2016), devoid of any known 7-dehydroxylating microbial activity, at steady-state (without prior antibiotic treatment) are susceptible or resistant to CDI. We compared this new model to previously established CDI models including completely axenic (germ-free) mice (Pawlowski et al., 2010), antibiotic- (streptomycin- and clindamycin-) pretreated SPF mice (Buffie et al., 2011; Ng et al., 2013) and unmanipulated (and highly CDI resistant) SPF mice. All animals were infected with 10^3^ CFU of *C. difficile* spores of strain DH1916 (Ribotype 027; Burns et al., 2011) and sacrificed 72 h post infection. Quantification of *C. difficile* in feces at 6 and 24 h post infection, and in cecal content at 72 h post infection revealed high *C. difficile* colonization levels in germ-free, antibiotic-treated SPF and gnotobiotic sDMDMm2 mice (Figure 1A). In contrast, unmanipulated SPF animals, apart from the gastrointestinal transit of the inoculum detected around 6 h post-infection, showed no evidence for productive intestinal *C. difficile* colonization. In all CDI-susceptible mouse models that we tested, the levels of the intestinal inflammation markers lipocalin-2 (Figure 1B) and calprotectin (Figure 1C) were strongly elevated over the background levels measured in non-infected control mice and the CDI-resistant SPF mice. Large intestinal histopathology revealed marked mucosal inflammation in *C. difficile*-infected germ-free, antibiotic-treated SPF, and gnotobiotic sDMDMm2 animals, but not in the non-antibiotic-treated SPF mice (Figures 1D,E). Clindamycin-treated uninfected controls showed an elevated baseline histopathology of unknown etiology (see Figure 1D) compared to non-antibiotic-treated SPF mice. In summary, the sDMDMm2 CDI model is non-colonization-resistant against *C. difficile* and closely reproduces the colonization dynamics and pathogenesis of the widely used antibiotic-pretreatment mouse models.

### Comprehensive profiling of bile acid metabolome associated with primary and antibiotic-induced CDI susceptibilities

**Figure 2:**
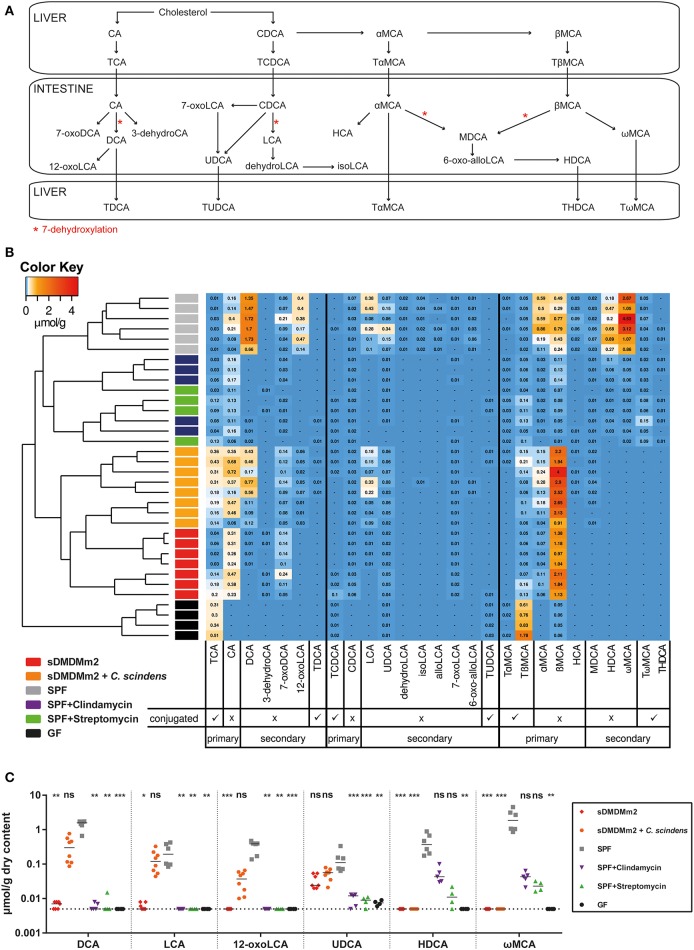
**Pre-colonization with ***C. scindens*** partially restores a SPF-like bile acid profile**. SPF mice were pre-treated with streptomycin (*n* = 4) or clindamycin (*n* = 5) 24 h before sacrifice; sDMDMm2 mice pre-colonized 7 days before sacrifice with *C. scindens* (*n* = 8), unmanipulated SPF (*n* = 6), sDMDMm2 (*n* = 7), and germ-free (*n* = 4) animals were used as controls. Cecal content was aseptically removed and processed for bile acid metabolome quantification. Data was assembled from three individual experiments. **(A)** Schematic diagram of murine bile acid metabolism. Adapted from Zhang et al. (2012) and Song et al. (2011). **(B)** Heatmap analysis constructed from bile acid LC-MS/MS measurements. Values indicate measured bile acid concentrations in μmol/g dry weight of cecal content. **(C)** Analysis of individual secondary bile acids elevated in sDMDMm2 + *C. scindens* mice vs. sDMDMm2 mice. Statistical analysis used the Kruskal–Wallis test with Dunn's post-test to compare individual bile acids of the different groups against SPF. Each symbol represents one individual. Bars indicate medians. Dotted lines indicate lower limit of detection. ns, not statistically significant (*p* ≥ 0.05); ^*^p < 0.05; ^**^p < 0.01; ^***^p < 0.001. TCA, taurocholic acid; CA, cholic acid; DCA, deoxycholic acid; 3-dehydroCA, 3-dehydrocholic acid; 7-oxoDCA, 7-oxodeoxycholic acid; 12-oxoLCA, 12-oxolithocholic acid; TDCA, taurodeoxycholic acid; TCDCA, taurochenodeoxycholic acid; CDCA, chenodeoxycholic acid; LCA, lithocholic acid; UDCA, ursodeoxycholic acid; dehydroLCA, dehydrolithocholic acid; isoLCA, iso-lithocholic acid; alloLCA, allo-lithocholic acid; 7-oxoLCA, 7-oxolithocholic acid; TUDCA, tauroursodeoxycholic acid; TαMCA, tauro-α-muricholic acid; TβMCA, tauro-β-muricholic acid; αMCA, α-muricholic acid; βMCA, β-muricholic acid; HCA, hyocholic acid; MDCA, murodeoxycholic acid; 6-oxo-allo-LCA, 6-oxo-allo-lithocholic acid; HDCA, hyodeoxycholic acid; ωMCA, ω-muricholic acid; TωMCA, tauro-ω-muricholic acid; THDCA, taurohyodeoxycholic acid.

The available metagenomic information of the Oligo-MM^12^ consortium (Lagkouvardos et al., 2016) predicts that the Oligo-MM^12^ microbiota is able to deconjugate bile acids *in vivo*, but that none of the 12 strains harbors the metabolic pathway for bile acid 7α-dehydroxylation to generate secondary bile acids. To test this hypothesis and to identify discrete bile acid metabolome profiles associated with CDI susceptibility in the different models we carried out targeted UHPLC-HRMS based metabolomics. Instead of *C. difficile* inoculation, we sacrificed cohorts of germ-free, streptomycin-treated SPF, clindamycin-treated SPF, unmanipulated SPF, and gnotobiotic sDMDMm2 mice and quantified 36 bile acid species (nine of which were below the minimal detectable concentrations in all samples and omitted from the analysis; see Figure 2A and Table S1) in cecum content extracts. An additional group included in this analysis were sDMDMm2 animals that had been colonized for 7 days with *C. scindens*, which we hypothesized would increase the large intestinal concentrations of secondary bile acids *in vivo*.

Heat map analysis with unsupervised hierarchical clustering (Canberra distance) of individual mice based on the quantities of each bile acid derivative showed distinct bile acid profiles that grouped the animals according to their experimental treatment (Figure 2B). Germ-free mice were distinguished from all microbially colonized groups by the near-complete absence of deconjugated primary as well as secondary bile acids. Bile acid deconjugation by bile acid hydrolases is a common intestinal microbial metabolic activity, and correspondingly absent from germ-free animals, but evident in all microbially colonized animal groups tested in this experiment. Both antibiotic-treated CDI infection models cluster with the untreated SPF animals but were characterized by an overall reduction of all bile acids, specifically a marked decrease in secondary bile acids and nearly undetectable amounts of DCA and LCA in particular. Germ-free animals, as well as sDMDMm2 + *C. scindens* and to some extent the sDMDMm2 gnotobiotes accumulated higher cecal concentrations of the conjugated primary bile acid TCA than SPF mice. Similar to the other CDI-susceptible groups, Oligo-MM^12^-colonized sDMDMm2 mice lacked most secondary bile acids. The exception was 7-oxodeoxycholic acid (7-oxoDCA), which remained at levels equal or higher than found in SPF control mice. Hence, CDI-susceptible animals were distinguished from CDI-resistant animals by reduced levels of the six secondary bile acid species DCA, 12-oxolithocholic acid (12-oxoLCA), LCA, ursodeoxycholic acid (UDCA), hyodeoxycholic acid (HDCA), and ωMCA (Figure 2C). Finally, of these six secondary bile acid species the levels of four, DCA, LCA, UDCA, and 12-oxoLCA were fully or partially restored to the concentrations found in SPF control mice by the additional colonization of sDMDMm2 mice with *C. scindens*.

### Intestinal colonization of sDMDMm2 mice with *C. scindens* and bile acid profile normalization correlate with transient attenuation of CDI

**Figure 3:**
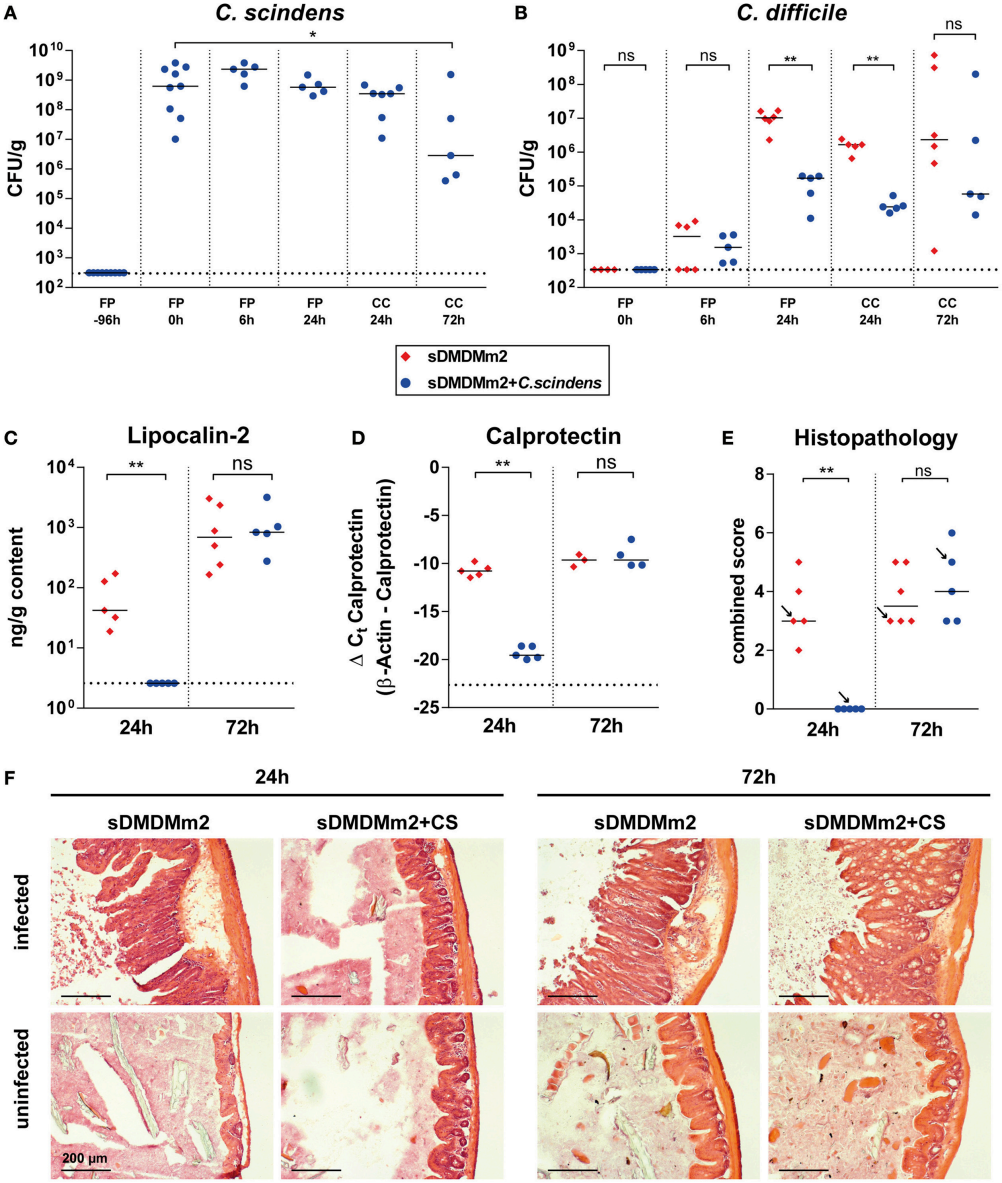
**Addition of ***C. scindens*** to sDMDMm2 can partially protect from ***C. difficile*** infection**. sDMDMm2 animals were pre-colonized by gavage with 10^9^ CFU *C. scindens* 96 h before infection with 10^3^ CFU *C. difficile* DH1916 (blue circles). sDMDMm2 animals (red diamonds) served as control. Data is combined from two independent experiments with endpoints at 24 h (*n* = 5 sDMDMm2 and *n* = 5 sDMDM + *C. scindens*, only data from endpoint shown) and 72 h (*n* = 6 sDMDMm2 and *n* = 5 sDMDM + *C. scindens*). **(A)** CFU of *C. scindens* over the course of the infection in pre-colonized mice. **(B)** Comparison of *C. difficile* CFU between sDMDMm2 and sDMDMm2 + *C. scindens* over the course of infection. Uninfected controls are not depicted. **(C)** Lipocalin-2 concentration measured by ELISA in cecal content at 24 and 72 h post infection, respectively. **(D)** Mucosal calprotectin expression in cecal tissue measured by qPCR at 24 and 72 h post infection, respectively. Values show the ΔC_*t*_ derived from measured values of calprotectin with β-actin as control. **(E)** Histopathological evaluation of H&E stained sections of cecum tissue at time of necropsy (24 or 72 h). Uninfected controls are not depicted. Arrows indicate the representative mice depicted in **(F)**. **(F)** H&E stained histological sections of cecum sampled at 24 and 72 h post infection. FP, Fecal pellet; CC, Cecal content; L, Lumen; Scale bars: 200 μm. Statistical analyses in **(B–E)** used Mann-Whitney-U tests; Each symbol represents one individual. Bars indicate medians. Dotted lines indicate lower limit of detection. ns, not statistically significant (*p* ≥ 0.05); ^*^p < 0.05; ^**^p < 0.01.

**Figure 4:**
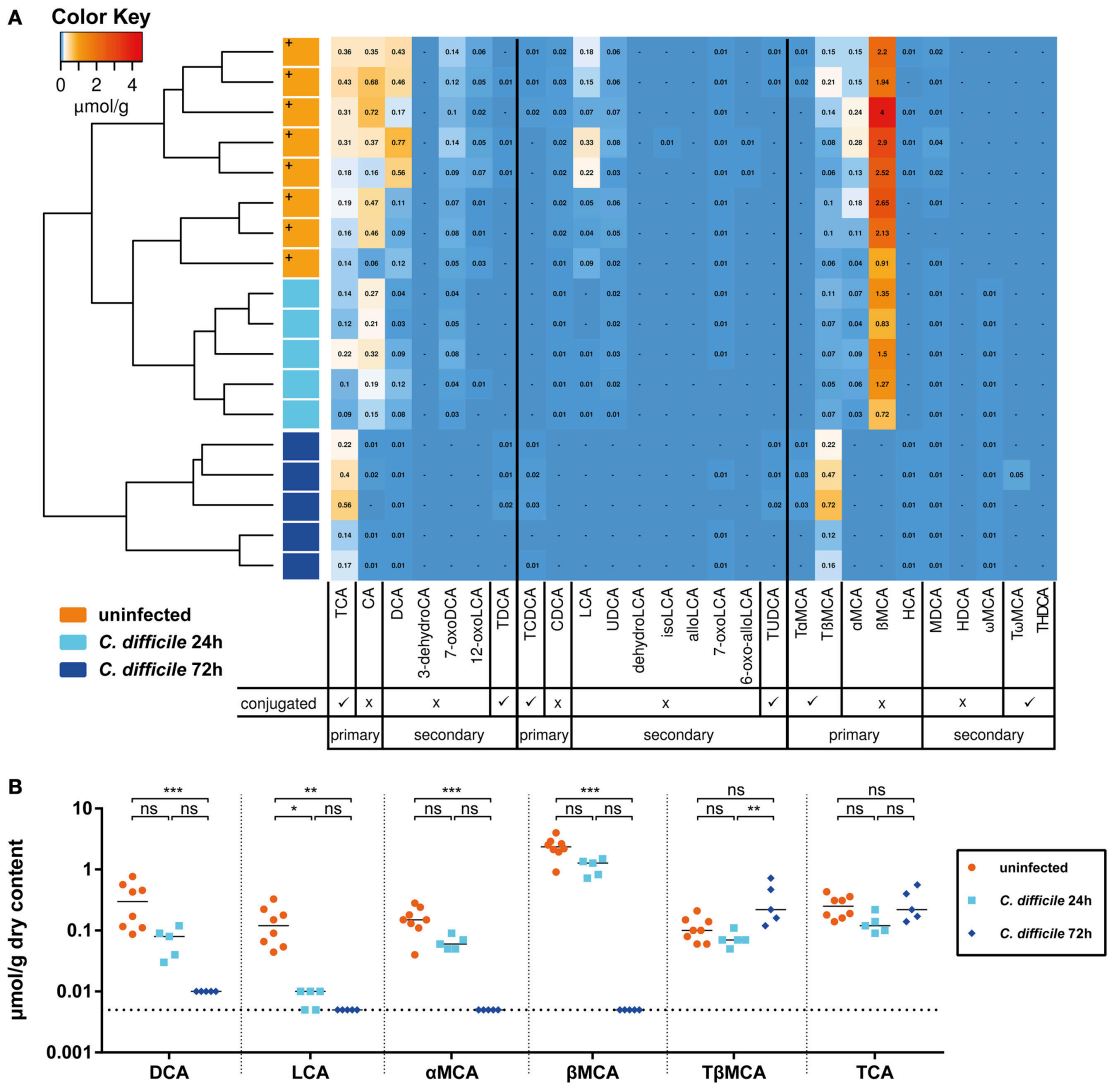
**Infection of sDMDMm2 + ***C. scindens*** mice with ***C. difficile*** leads to decrease in secondary and deconjugated primary bile acids**. Cecal contents of sDMDMm2 + *C. scindens* mice infected with *C. difficile* used in Figure 3 were used to quantify bile acid measurements. Measurements from uninfected control mice (colonized with *C. scindens* for 7 days) from Figure 2 were included as comparison (orange symbols, indicated by plus sign). **(A)** Heatmap analysis constructed from bile acid LC-MS/MS measurements. Values indicate measured bile acid concentrations in μmol/g dry weight of cecal content. **(B)** Analysis of individual bile acids in sDMDMm2 + *C. scindens* mice infected by *C. difficile* over time. Statistical analysis used the Kruskal-Wallis test with Dunn's post-test to compare individual bile acids between the groups. Each symbol represents one individual. Bars indicate medians. Dotted lines indicate lower limit of detection. ns, not statistically significant (*p* ≥ 0.05); ^*^p < 0.05; ^**^p < 0.01; ^***^p < 0.001. TCA, taurocholic acid; CA, cholic acid; DCA, deoxycholic acid; 3-dehydroCA, 3-dehydrocholic acid; 7-oxoDCA, 7-oxodeoxycholic acid; 12-oxoLCA, 12-oxolithocholic acid; TDCA, taurodeoxycholic acid; TCDCA, taurochenodeoxycholic acid; CDCA, chenodeoxycholic acid; LCA, lithocholic acid; UDCA, ursodeoxycholic acid; dehydroLCA, dehydrolithocholic acid; isoLCA, iso-lithocholic acid; alloLCA, allo-lithocholic acid; 7-oxoLCA, 7-oxolithocholic acid; TUDCA, tauroursodeoxycholic acid; TαMCA, tauro-α-muricholic acid; TβMCA, tauro-β-muricholic acid; αMCA, α-muricholic acid; βMCA, β-muricholic acid; HCA, hyocholic acid; MDCA, murodeoxycholic acid; 6-oxo-allo-LCA, 6-oxo-allo-lithocholic acid; HDCA, hyodeoxycholic acid; ωMCA, ω-muricholic acid; TωMCA, tauro-ω-muricholic acid; THDCA, taurohyodeoxycholic acid.

Next, we analyzed how colonization with *C. scindens* and the concomitant changes in secondary bile acid concentrations correlated with the course of CDI. For this purpose a cohort of sDMDMm2 mice was infected with *C. difficile* 4 days after association with *C. scindens*. One sub-cohort was sacrificed 24 h after infection (day 5 after *C. scindens* colonization), the other 72 h after infection (day 7 after *C. scindens* colonization). At the time of sacrifice, we quantified *C. scindens* (Figure 3A) and *C. difficile* densities (Figure 3B), cecal mucosal pathology (by histopathological scoring, luminal lipocalin-2 protein, and mucosal calprotectin mRNA quantification; Figures 3C–F), and cecal luminal bile acid composition (by UHPLC-HRMS in sDMDM + *C. scindens* mice; Figure 4). At the time of infection, *C. scindens* had reached levels of 10^8^ −10^9^ CFU/g feces (Figure 3A). The *C. scindens*-associated mice at 24 h post infection showed partial resistance against *C. difficile* colonization compared to controls (Figures 3B–F), with significantly reduced fecal and cecal *C. difficile* loads and absence of marked cecal pathology. This partially protected phenotype was associated with the presence of secondary bile acid DCA in the cecal contents (Figures 4A,B, light blue symbols; reference dataset of uninfected sDMDMm2 mice colonized with *C. scindens* for 7 days from Figure 2 is shown for comparison; orange symbols, indicated by plus sign). However, we observed a complete loss of protection by 72 h post infection, when large intestinal colonization levels of *C. difficile* and cecal pathology were no longer significantly different from controls (Figures 3B–F). Median *C. scindens* densities were decreased at this time point compared to before infection with *C. difficile*. The loss of protection was further associated with a near-complete absence of microbial bile acid transformation products in cecum contents in these animals (Figures 4A,B; dark blue symbols). The concentrations of secondary and primary deconjugated bile acids were near or below detection limits, very similar to the bile acid profiles observed in germ-free animals (see Figure 2B). These data show that colonization with *C. scindens* impacts the early colonization dynamics but could not fully prevent *C. difficile* colonization and pathogenesis. This is consistent with the previously reported effects of DCA and LCA in inhibition of vegetative growth (Sorg and Sonenshein, 2008; Buffie et al., 2014) described *in vitro* and *ex vivo*. The association between CDI-induced intestinal pathology and the abolition of intestinal microbial bile acid transformations (deconjugation and 7-dehydroxylation) to germ-free mouse-like levels prompted us to analyze the effect of CDI on the large intestinal Oligo-MM^12^ microbiota composition.

### Effects of *C. scindens* and *C. difficile* association on Oligo-MM^12^ composition

**Figure 5:**
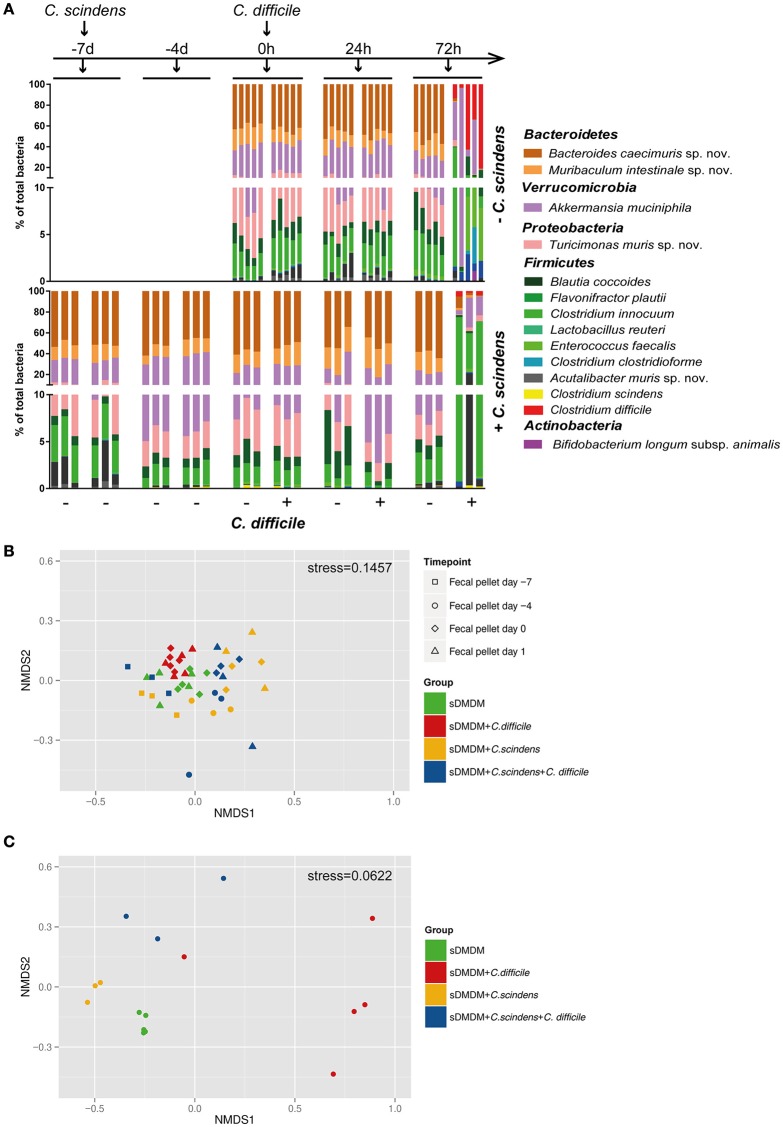
**Addition of ***C. scindens*** leads only to small changes in the composition of the Oligo-MM^**12**^ microbiota. (A)** qPCR based microbiota analysis of all 12 Oligo-MM strains as well as *C. difficile* and *C. scindens* from fecal pellets (−7 days, −4 days, 0 h, 24 h) or cecal content (72 h). Top graph: *n* = 5 sDMDMm2 and *n* = 5 sDMDMm2 + *C. difficile* animals. Bottom Graph: *n* = 3 sDMDMm2 + *C. scindens* (pre-colonized for 7 days) and *n* = 3 sDMDMm2 + *C. scindens* + *C. difficile* animals (pre-colonized for 7 days before infection of 3 days). Y-Axis is depicted as two-segment axis. **(B)** Non-parametric multidimensional scaling of the Oligo-MM^12^ microbiota data shown in **(A)**, analyzed from fecal pellets from time points *t* = −7 days (before *C. scindens* colonization), *t* = −4 days, *t* = 0 h (before *C. difficile* infection), and *t* = 24 h (without using the qPCR data of *C. difficile* or *C. scindens*). **(C)** Non-parametric multidimensional scaling of the Oligo-MM^12^ microbiota data shown in **(A)**, analyzed from cecal contents from timepoint *t* = 72 h (without using the qPCR data of *C. difficile* or *C. scindens*).

To analyze the impact of CDI on sDMDMm2 microbiota composition, sDMDMm2 mice were pre-colonized for 7 days with *C. scindens* followed by infection with *C. difficile* for 72 h (or uninfected controls). For comparison, we analyzed non-pretreated sDMDMm2 mice infected with *C. difficile* for 72 h (and uninfected controls). We analyzed the bacterial consortium composition over time in the feces and at the endpoint in cecal contents using an established 16S rRNA-based TaqMan qPCR method (see materials and Methods; Brugiroux et al., 2016) including probes for all Oligo-MM^12^ species as well as *C. scindens* and *C. difficile* (Figure 5A, Figure S1). We quantified the fecal (−7 days, −4 days, 0 h, 24 h post infection) as well as cecal (72 h post infection) Oligo-MM^12^ composition *C. scindens*-associated and non-associated mice to determine changes in response to *C. scindens* colonization alone, as well as in response to *C. difficile* infection (Figure 5A, Figure S1). Non-parametric multidimensional scaling (using the metaMDS function of the vegan package in R) with the qPCR data of the 12 bacterial strains of the Oligo-MM^12^ microbiota (excluding the quantities of *C. difficile* and *C. scindens*) of all fecal samples showed that the colonization of sDMDMm2 animals with *C. scindens* led to minor changes in Oligo-MM^12^ composition (compare yellow and green symbols in Figure 5B). These changes were mainly driven by variability in the densities of *C. clostridioforme* and *A. muris* sp. nov. (Figure S1) over the time of the colonization with *C. scindens* and were not statistically significant.

As in non-pretreated sDMDMm2 mice, sDMDMm2 + *C. scindens* associated mice after 72 h of infection with *C. difficile* showed a severely perturbed cecal microbial consortium, whose species composition differed significantly from uninfected control animals (Figures 5A,C, *p* = 0.008 for sDMDMm2; *p* = 0.001 for sDMDMm2 + *C. scindens*). Pre-colonization with *C. scindens* had no statistically significant influence on this outcome (Figure 5C, *p* = 0.145, compare blue and red circles). The observed shift in microbiota composition was associated with a decreased density of *C. scindens*, as well as most of the Oligo-MM^12^ bacterial species, reflected in a decreased total bacterial density (Figure S1).

## Discussion

For the presented study we utilized a defined mouse model associated with a stable low-complexity microbiota that represents the main phyla of the murine intestinal microbiota. It has recently been used to study colonization resistance against the intestinal pathogen *Salmonella enterica* serovar Typhimurium (Brugiroux et al., 2016), and for the study of the metabolism of the facultative intestinal commensal species *E. coli* K-12 in the intestinal lumen and mucus layer (Li et al., 2015). In both examples, it was important for the microbiological process under study to be tested in context with a reproducible and defined host–microbial metabolic background. The host–microbiota background metabolism was also important in our study: the Oligo-MM^12^ consortium carried out primary bile acid deconjugation, a prerequisite for 7α-dehydroxylation.

We detected only minor changes in the Oligo-MM^12^ microbiota composition following the addition of *C. scindens*, including decreases in *C. clostridioforme*, and *A. muris* sp. nov. To what extent these changes are attributable to the increased concentrations of secondary bile acids or direct competition with *C. scindens* remains unknown.

The intestinal bile acid metabolome is closely linked to the resistance of the gastrointestinal tract against CDI. It has been shown recently that CDI treatment by fecal microbiota transplantation therapy not only leads to an increase in gut microbial diversity, but also reestablishes a bile acid metabolome that closely mimics that of the stool donor (Weingarden et al., 2013, 2016). Our data corroborate the association of *C. scindens*-produced secondary bile acids DCA and LCA with CDI resistance *in vivo*, likely related to inhibition of spore outgrowth or vegetative growth (see Figure 2; Buffie et al., 2014).

Formal proof of causality of *C. scindens*-produced secondary bile acids in inhibition of CDI remains a challenge for as long as *C. scindens* and related species cannot be genetically manipulated. This technical limitation currently prohibits the study of isogenic *bai* gene mutants. Thus, we currently cannot rule out the possibility that the association between *C. scindens* colonization and transient inhibition of *C. difficile* colonization and pathogenesis is independent of secondary bile acid production by *C. scindens*, but rather the result of other modes of direct or indirect interference between both species.

In the sDMDMm2 mouse model, *C. scindens* alone clearly has only a rather discrete, transient protective effect, but confers no colonization resistance comparable to that conferred by a much more diverse SPF microbiota. Full colonization resistance may require a more diverse microbial consortium including additional microbial species that compete for nutrients such as simple sugars (Ng et al., 2013), metals (Zackular et al., 2016), or other resources required for intestinal colonization. The importance of other species has also been demonstrated by the finding that therapeutic intestinal microbial cocktails may not need to include 7-dehydroxylating species to be effective (Lawley et al., 2012).

The analysis of bile acid composition in the *C. scindens*-colonized sDMDMm2 mice following 24 and 72 h of infection revealed that the onset of *C. difficile*-induced pathology was associated with the severe disruption of microbiota-mediated bile acid transformations. Whilst at 24 h post infection, when the mice were still significantly protected from the infection, bile acid 7α-dehydroxylation and bile acid deconjugation were still evident, at 72 h post infection the bile acid metabolome was reduced to a germ-free-like status. This was associated with a significant compositional perturbation of the Oligo-MM^12^ consortium at 72 h post infection (Figure 5).

In conventional mice, unlike humans, LCA is only a minor secondary bile acid species, whereas major murine secondary bile acids, absent in humans, are the MCA derivatives HDCA, and ωMCA, which are microbially generated from the primary bile acids αMCA and βMCA. The mouse-specific primary bile acids αMCA and βMCA were shown by others to be inhibitors of *C. difficile* spore germination and vegetative growth at the relevant concentrations found in mice (Francis et al., 2013b). However, in our experiments we could not observe any indication of a protective effect in GF (harboring increased TβMCA levels) or sDMDMm2 mice (harboring increased amounts of βMCA) over the antibiotic-treated mouse models (harboring lower levels of both compounds).

As reported previously (Sacquet et al., 1984), we found that, like other 7α-dehydroxylating human isolates, *C. scindens* ATCC35704 was unable to transform the mouse-specific primary MCAs (see Figure 2), whereas murine isolates have been shown to do so (Eyssen et al., 1999). In keeping with these older reports, our measurements showed that *C. scindens* association did not significantly increase the concentrations of the mouse-specific secondary bile acids HDCA or ωMCA. There is previous evidence that links ωMCA to inhibition of germination and vegetative cell growth of *C. difficile* (Francis et al., 2013b). This may have limited the CDI-inhibitory effect of human-derived *C. scindens* in the murine system. We hypothesize, that colonization of sDMDMm2 mice with a mouse-derived 7α-dehydroxylating bacterium may be able to reestablish a bile acid metabolome that is even closer to the bile acid metabolome of conventional SPF mice. Such a more complete bile acid metabolome may confer higher resistance against CDI in mice, but would not have a direct correlate in the human system.

In future efforts, the further modular re-constitution of microbial bile acid transformations in gnotobiotic models will not necessarily be a mere reductionist approach in comparison to conventional unstandardized SPF animals. For example, the human liver, unlike its murine counterpart, is incapable of re-hydroxylating recirculating secondary bile acids at the seven position, which in some human individuals can lead to the accumulation of pathologically elevated levels of DCA and LCA, associated with enteropathy, and increased colon cancer risk (Barrasa et al., 2012). The detoxification pathway of DCA and LCA by epimerization at the three position, generating the 3β-hydroxy(iso)-bile acids isoDCA and isoLCA, has recently been elucidated as a novel microbially encoded pathway (Devlin and Fischbach, 2015). Iso-bile acids are major bile acid species in some humans but absent in others, and were shown to be generated by the human commensal bacterium *Ruminococcus gnavus*. Although our analysis so far did not include isoDCA, we did not measure detectable levels of the corresponding LCA derivative isoLCA in the SPF control mice, suggesting that commonly used SPF mice (at least from our supplier) may lack these organisms and therefore the possibility to study this important detoxification pathway in the mouse model. Thus, for the study of the human pathological condition associated with deficiency of this bile acid detoxifying transformation, a combination of adding a 3-epimerizing bacterial species to the microbiota and the genetic engineering of the murine liver metabolism (by knockout of the 7α-rehydroxylation pathway) could provide a useful disease model that extends the microbiome repertoire of conventional mice.

The presented work represents a first step toward the normalization of host–microbial bile acid co-metabolism in a gnotobiotic mouse model that is well-established (Li et al., 2015; Brugiroux et al., 2016). The rational amendment of additional microbial metabolic modules in the future will provide further refined models for the study of host–microbial interactions using defined microbial consortia and may pave the way toward better-standardized animal models.

## Author contributions

Conceived the experiments: NS, LD, SK, NM, RB, and SH. Performed *in vivo* experiments: NS and MT. Evaluated the histopathology: CS. Performed bile acid measurements and analysis: LD, LM, and NS. Developed and analyzed Oligo-MM12 consortium: MB, NS, SB, KM, and BS. Wrote the paper: NS and SH. All authors read and commented on the final manuscript.

## Funding

SH, RB, and NM received funding for this work from the Swiss National Science Foundation (Sinergia grant CRSII3_147603). BS received funding from the BMBF (Medizinische Infektionsgenomik), the DFG Priority program SPP1656, the German Center for Infection Research (DZIF), and the Centre for Gastrointestinal Microbiome Research (CEGIMIR). KM is supported by a grant from the SNSF (SNSF310030_134902) and the European Research Council (ERC, FP/2007–2013) Agreement no 281785. The funders had no role in study design, data collection and interpretation, or the decision to submit the work for publication.

### Conflict of interest statement

The authors declare that the research was conducted in the absence of any commercial or financial relationships that could be construed as a potential conflict of interest.
