# Supplementary material for: Functional Intestinal Bile Acid 7α-Dehydroxylation by Clostridium scindens Associated with Protection from Clostridium difficile Infection in a Gnotobiotic Mouse Model
Source: Front Cell Infect Microbiol. 2016 Dec 20;6:191. doi: 10.3389/fcimb.2016.00191 (PMC5168579; doi:10.3389/fcimb.2016.00191)
Supplement: Supplementary file 1 [file Table1.PDF]

Table S1. Bile acids identified and quantified by UHPLC-HRMS.

Name	Abbreviation	R_t (min)	$[M-H]^-$ theor.	$[M-H]^-$ measured	Mass Error (ppm)
**Dehydrolithocholic acid**[b]	dehydroLCA	18.54	373.2748	373.2754	-1.6
**Allolithocholic acid**[b]	alloLCA	17.72	375.2905	375.2899	1.6
**Isolithocholic acid**[b]	isoLCA	17.89	375.2905	375.2910	-1.3
**Lithocholic acid**[b]	LCA	18.36	375.2905	375.2909	-1.1
**6-oxo-allolithocholic acid**[b]	6-oxo-alloLCA	13.13	389.2697	389.2697	0.0
**7-oxolithocholic acid**[b]	7-oxoLCA	13.44	389.2697	389.2705	-2.1
**12-oxolithocholic acid**[b]	12-oxoLCA	13.72	389.2697	389.2711	-3.6
**Murideoxycholic acid**[b]	MDCA	10.06	391.2854	391.2856	-0.5
**Ursodeoxycholic acid**[c]	UDCA	11.73	391.2854	391.2858	-1.0
**Hyodeoxycholic acid**[d]	HDCA	12.34	391.2854	391.2860	-1.5
**Chenodeoxycholic acid**[b]	CDCA	16.32	391.2854	391.2859	-1.3
**Deoxycholic acid**[b]	DCA	16.50	391.2854	391.2858	-1.0
**3-deoxycholic acid**[a,b]	3-DCA	17.50	391.2854	391.2850	1.0
**deuterated Chenodeoxycholic acid**[b]	CDCA-D4	16.30	395.3105	395.3111	-1.5
**Deuterated Deoxycholic acid**[b]	DCA-D4	16.49	395.3105	395.3112	-1.8
**7-oxodeoxycholic acid**[b]	7-oxoDCA	8.22	405.2647	405.2649	-0.5
**3-dehydrocholic acid**[b]	3-dehydroCA	10.56	405.2647	405.2652	-1.2
**ω-muricholic acid**[b]	ωMCA	6.36	407.2803	407.2818	-3.7
**α-muricholic acid**[b]	αMCA	7.17	407.2803	407.2817	-3.4
**β-muricholic acid**[b]	βMCA	8.01	407.2803	407.2810	-1.7
**Hyocholic acid**[b]	HCA	10.35	407.2803	407.2805	-0.5
**Cholic acid**[d]	CA	11.75	407.2803	407.2807	-2.0
**Glycolithocholic acid**[a,b]	GLCA	16.54	432.3119	432.3123	-0.9
**Glycoursodeoxycholic acid**[a,b]	GUDCA	9.34	448.3068	448.3072	-0.9
**Glycohyodeoxycholic acid**[a,b]	GHDCA	9.73	448.3068	448.3067	0.2
**Glycochenodeoxycholic acid**[a,c]	GCDCA	13.27	448.3068	448.3071	-0.7
**Glycodeoxycholic acid**[a,b]	GDCA	14.09	448.3068	448.3072	-0.9
**Glycocholic acid**[a,b]	GCA	10.35	464.3018	464.3036	-3.9
**Taurolithoicholic acid**[a,b]	TLCA	16.38	482.2946	482.2959	-2.7
**Tauroursodeoxycholic acid**[b]	TUDCA	10.21	498.2895	498.2896	-0.2
**Taurohyodeoxycholic acid**[b]	THDCA	10.60	498.2895	498.2900	-1.0
**Taurochenodeoxycholic acid**[c]	TCDCA	14.11	498.2895	498.2893	0.4
**Taurodeoxycholic acid**[c]	TDCA	14.96	498.2895	498.2887	1.6
**Tauro-ω-muricholic acid**[b]	TωMCA	4.69	514.2844	514.2859	-2.9
**Tauro-α-muricholic acid**[b]	TαMCA	5.21	514.2844	514.2825	3.7
**Tauro-β-muricholic acid**[b]	TβMCA	5.46	514.2844	514.2850	-1.2
**Taurohyocholic acid**[a,b]	THCA	9.18	514.2844	514.2850	-1.2
**Taurocholic acid**[c]	TCA	10.99	514.2844	514.2841	0.6

[a] Not detected in any samples
[b] from Steraloids, Inc. (Newport, RI, USA)
[c] from Sigma-Aldrich (Switzerland)
[d] from VWR (Switzerland)
