# Supplementary material for: Functional Intestinal Bile Acid 7α-Dehydroxylation by Clostridium scindens Associated with Protection from Clostridium difficile Infection in a Gnotobiotic Mouse Model
Source: Front Cell Infect Microbiol. 2016 Dec 20;6:191. doi: 10.3389/fcimb.2016.00191 (PMC5168579; doi:10.3389/fcimb.2016.00191)
Supplement: Supplementary file 2 [file Table2.PDF]

**Table S2.** Primers used for Quantitative PCR of bacterial 16S rRNA genes in this study

| Designation | Sequence (5´ - 3´) | Specificity | Detection limit (qPCR) | Reference |
|---|---|---|---|---|
| Isol46_Exonucl.2_fwd | CGGATCGTAAAGCTCTGTTGTAAG | I46 | 125 copies/5ng template gDNA | Brugiroux et al. |
| Isol46_Exonucl.3_rev | GCTACCGTCACTCCCATAGCA | | | |
| Probe3_Isol46 | FAM-AAGAACGGCTCATAGAGG-BHQ1 | | | |
| Isol49_Exonucl._fwd | GCACTGGCTCAACTGATTGATG | I49 | 248 copies/5ng template gDNA | Brugiroux et al. |
| Isol49_Exonucl._rev | CCGCCACTCACTGGTGATC | | | |
| Probe_Isol49 | HEX-CTTGCACCTGATTGACGA-BHQ1 | | | |
| YL58_Exonucl._fwd | GAAGAGCAAGTCTGATGTGAAAGG | YL58 | 141 copies/5ng template gDNA | Brugiroux et al. |
| YL58_Exonucl._rev | CGGCACTCTAGAAAAACAGTTTCC | | | |
| Probe_YL58 | FAM-TAACCCCAGGACTGCAT-BHQ1 | | | |
| YL27_Exonucl.2_fwd | TCAAGTCAGCGGTAAAAATTCG | YL27 | 4 copies/5ng template gDNA | Brugiroux et al. |
| YL27_Exonucl.2_rev | CCCACTCAAGAACATCAGTTTCAA | | | |
| Probe2_YL27 | HEX-CAACCCCGTCGTGCC-BHQ1 | | | |
| YL31_Exonucl.2_fwd | AGGCGGGATTGCAAGTCA | YL31 | 4 copies/5ng template gDNA | Brugiroux et al. |
| YL31_Exonucl.3_rev | CCAGCACTCAAGAACTACAGTTTCA | | | |
| Probe2_YL31 | FAM-CAACCTCCAGCCTGC-BHQ1 | | | |
| YL32_Exonucl.2_fwd | AATACCGCATAAGCGCACAGT | YL32 | 2 copies/5ng template gDNA | Brugiroux et al. |
| YL32_Exonucl.2_rev | CCATCTCACACCACCAAAGTTTT | | | |
| Probe2_YL32 | HEX- CGCATGGCAGTGTGT-BHQ1 | | | |
| KB1_Exonucl._fwd | CTTCTTTCCTCCCGAGTGCTT | KB1 | 25 copies/5ng template gDNA | Brugiroux et al. |
| KB1_Exonucl._rev | CCCCTCTGATGGGTAGGTTACC | | | |
| Probe_KB1 | FAM-CACTCAATTGGAAAGAGGAG-BHQ1 | | | |

| **Designation** | **Sequence (5´ - 3´)** | **Specificity** | **Detection limit (qPCR)** | **Reference** |
|---|---|---|---|---|
| YL2_Exonucl._ fwd | GGGTGAGTAATGCGTGACCAA | YL2 | 13 copies/5ng template gDNA | Brugiroux et al. |
| YL2_Exonucl._ rev | CGGAGCATCCGGTATTACCA | | | |
| Probe_YL2 | HEX-CGGAATAGCTCCTGGAAA-BHQ1 | | | |
| KB18_Exonucl.2_fwd | TGGCAAGTCAGTAGTGAAATCCA | KB18 | 1 copies/5ng template gDNA | Brugiroux et al. |
| KB18_Exonucl.2_rev | TCACTCAAGCTCGACAGTTTCAA | | | |
| Probe2_KB18 | FAM-CTTAACCCATGAACTGC-BHQ1 | | | |
| YL44_Exonucl._ fwd | CGGGATAGCCCTGGGAAA | YL44 | 2 copies/5ng template gDNA | Brugiroux et al. |
| YL44_Exonucl._ rev | GCGCATTGCTGCTTTAATCTTT | | | |
| Probe_YL44 | HEX-TGGGATTAATACCGCATAGTA-BHQ1 | | | |
| YL45_Exonucl._ fwd | AGACGGCCTTCGGGTTGTA | YL45 | 2 copies/5ng template gDNA | Brugiroux et al. |
| YL45_Exonucl._ rev | CGTCATCGTCTATCGGTATTATCAA | | | |
| Probe_YL45 | FAM-ACCACTTTTGTAGAGAACGA-BHQ1 | | | |
| Isol48_Exonucl._fwd | GGCAGCATGGGAGTTTGCT | I48 | 4 copies/5ng template gDNA | Brugiroux et al. |
| Isol48_Exonucl._rev | TTATCGGCAGGTTGGATACGT | | | |
| Probe_Isol48 | HEX-CAAACTTCCGATGGCGAC-BHQ1 | | | |
| C.diff_Exonucl._ fwd | TCGGTGCCGCAGCTAAC | *C. difficile* | 25 copies/5ng template gDNA | This study |
| C.diff_Exonucl._ rev | AGTTTCACTCTTGCGAGCGTACT | | | |
| Probe_C.difficile | HEX-CATTAAGTACTCCGCCTGGG-BHQ1 | | | |
| C.scin_Exonucl._fwd | TGCAAGCCAGATGTGAAAGC | *C. scindens* | 25 copies/5ng template gDNA | This study |
| C.scin_Exonucl._rev | AGCCACGCAGTTCCAAATG | | | |
| Probe_C.scindens | HEX-CTCAACCCCGGGACT-BHQ1 | | | |
| Univ_Exonucl.3_ fwd | TGCAYGGYYGTCGTCAGC | All bacteria | 2500 copies/5ng template gDNA | Brugiroux et al. |
| Univ_Exonucl.3_ rev | CRTCRTCCYCRCCTTCCTC | | | |
| Probe3_Univ | HEX-ARCGAGCGYAACCC-BHQ1 | | | |
