# Supplementary figures and images for: Functional Intestinal Bile Acid 7α-Dehydroxylation by Clostridium scindens Associated with Protection from Clostridium difficile Infection in a Gnotobiotic Mouse Model
Source: Front Cell Infect Microbiol. 2016 Dec 20;6:191. doi: 10.3389/fcimb.2016.00191 (PMC5168579; doi:10.3389/fcimb.2016.00191)

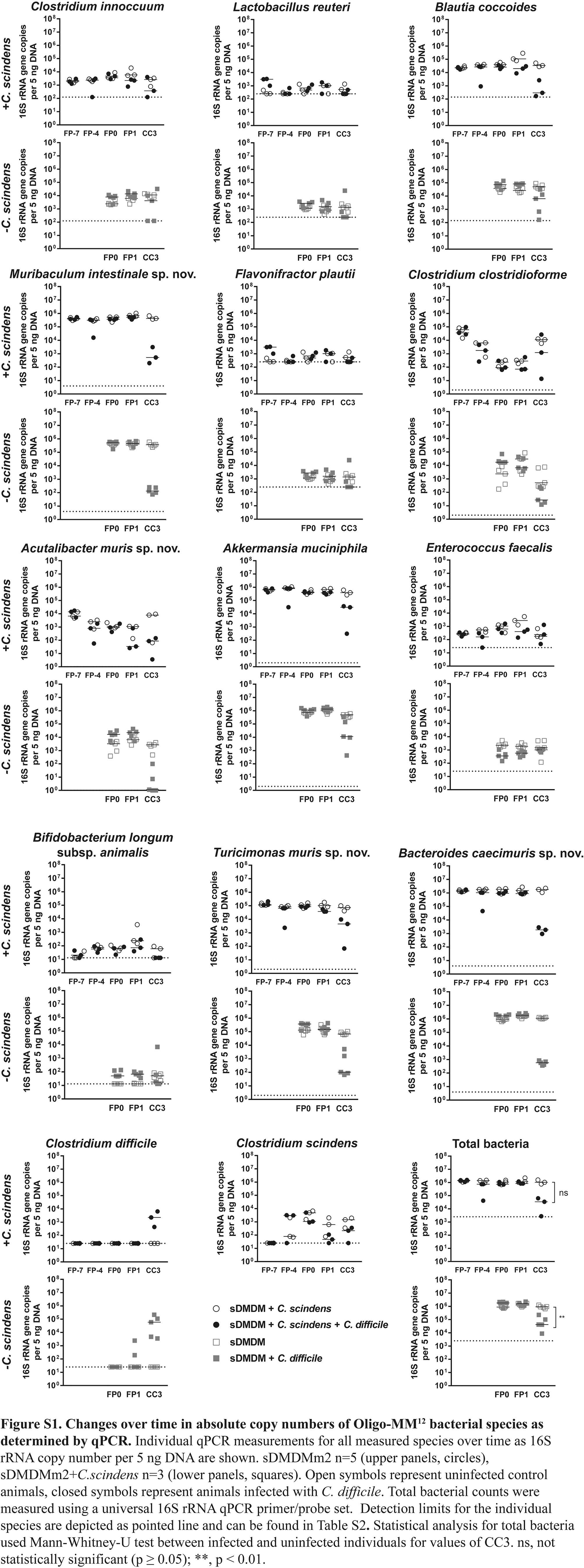

Supplement: Supplementary file 3 [file Image1.TIF]
